# Genetics of Pheochromocytomas and Paragangliomas Determine the Therapeutical Approach

**DOI:** 10.3390/ijms23031450

**Published:** 2022-01-27

**Authors:** Balazs Sarkadi, Eva Saskoi, Henriett Butz, Attila Patocs

**Affiliations:** 1 ELKH Hereditary Cancer Research Group, 1052 Budapest, Hungary; sarkadi.balazs.md@gmail.com (B.S.); butz.henriett@med.semmelweis-univ.hu (H.B.); 2 Department of Molecular Genetics, National Institute of Oncology, 1122 Budapest, Hungary; saskoi.eva@oncol.hu; 3 Department of Laboratory Medicine, Semmelweis University, 1085 Budapest, Hungary

**Keywords:** pheochromocytoma, paraganglioma, hereditary tumor, susceptibility genes, germline, somatic, metastatic, treatment, personalized medicine

## Abstract

Pheochromocytomas and paragangliomas are the most heritable endocrine tumors. In addition to the inherited mutation other driver mutations have also been identified in tumor tissues. All these genetic alterations are clustered in distinct groups which determine the pathomechanisms. Most of these tumors are benign and their surgical removal will resolve patient management. However, 5–15% of them are malignant and therapeutical possibilities for them are limited. This review provides a brief insight about the tumorigenesis associated with pheochromocytomas/paragangliomas in order to present them as potential therapeutical targets.

## 1. Introduction

Pheochromocytomas (PCC) and paragangliomas (PGL, together PPGL) are rare endocrine tumors originating from the chromaffin cells of the embryonic crest. PCC originate from the adrenal medulla whereas PGLs are extra-adrenally located in the abdomen, thorax, pelvis and neck. These tumors secrete catecholamines, except for the head and neck PGLs (HNPGL), which arise from the parasympathetic ganglia. The catecholamine excess prompts the classic, frequently paroxysmal symptoms such as headaches, palpitation, diaphoresis, syncope, abdominal pain, panic attacks, tremor, pallor and diarrhea. These symptoms are generally accompanied by hypertension and massive release of catecholamines can lead to cardiovascular complications as well [1,2]. Overall, approx. 40% of these tumors are developed due to inherited mutations of the various, growing number of PPGL genes which make PPGL the tumors with the highest susceptibility for inherited cancer syndrome [3,4]. Genetic testing is a cornerstone in patient management, affecting both treatment selection and clinical follow-up [5,6]. Although the tissue origin of these tumors is identical, the differences in tumorigenesis are remarkably distinct. Different molecular clusters are distinguished based on the pathogenesis [7]. Cluster 1 (which is further divided into two sub-clusters) represents the pseudohypoxia pathway with either germline or somatic mutation of *EGLN1*, *EGLN2*, *DLST*, *FH*, *IDH3B*, *MDH2*, *SDHA*, *SDHAF2*, *SDHB*, *SDHC*, *SDHD* or *VHL* and the exclusively postzygotic mutations of *EPAS1*, *IDH1* and *IDH2* genes. Cluster 2 stands for the kinase-signaling pathway group with germline or somatic mutations in *NF1*, *MAX*, *MERTK*, *MET*, *MYCN*, *RET* or *TMEM127* and mutations only reported in sporadic PPGL in *BRAF*, *HRAS* and *FGFR1* genes. Cluster 3 is a somatic cluster which represents the Wnt signaling pathway with *MAML3* fusion genes and the mutations in *CSDE1* gene. These clusters are eventually expanded by new candidate genes and novel potential clusters are emerging as well. Categorizing this heterogeneous, complex tumor group yields an opportunity for personalized patient management which results in a better clinical outcome. The diagnosis of malignant disease is established (radiologically or histologically) upon the presence of metastases, which is defined by the presence of chromaffin tissue where chromaffin cells are not typically found. The most common metastatic sites are lymph nodes, bone, liver and lungs [1]. Metastatic disease is rare, but almost half of the cases are associated with *SDHB* mutation-related PPGL, as patients with germline *SDHB* mutation have an overall 25% to 50% risk of developing metastatic disease [8,9]. The five-year overall survival ranges from 50% to 70% [10,11,12,13] The risk factors for metastatic disease additionally to germline *SDHB*-mutation comprise of noradrenergic or dopaminergic phenotype, size (>5 cm), and extra-adrenal location [14,15,16].

This brief review intends to give an insight into the specific molecular pathomechanism of the PPGL clusters and highlights the differences between them regarding clinical outcome and patient management. The clinical relevance of genetic background of PPGL tumors is also summarized to draw the attention to the existing genotype–phenotype correlations and their implication in clinical use.

## 2. Hereditary Factors

### 2.1. Pseudohypoxic Cluster

Pseudohypoxic cluster can be divided into two further subgroups: tricarboxylic acid (TCA) cycle-related and *VHL/EGLN1/EPAS1*-related mutations (Table 1).

Mutations in the genes encoding the several members of the tricarboxylic acid (TCA) cycle enzymes predispose to PPGL. The four succinate dehydrogenase subunits (*SDHA*, *SDHB*, *SDHC*, and *SDHD*), the SDH assembly factor (*SDHAF2*), fumarate hydratase (*FH*) and malate dehydrogenase 2 (*MDH2*) have been identified in about 12% to 16% of PPGL patients. Mutation in *SDHx* or *FH* genes, represent a high, 20–50% risk for metastatic disease [1,4,8,9,17,18]. Mutations in the genes encoding *SDHx* and *FH* result in increased levels of succinate and fumarate, respectively. Upon accumulation, these metabolites act as oncometabolites, altering cellular processes and promoting the survival and proliferation of tumor cells [19]. The elevated level of succinate or fumarate activates the pseudohypoxic pathway by inhibiting Hypoxia-Inducible Factor- (HIF) prolyl hydroxylases (and therefore stabilizing HIF under normoxic conditions) and promotes the hypermethylation of histone and DNA as competitive inhibitors of alpha-ketoglutarate-dependent dioxygenases. These include Jumonji domain-containing histone demethylases and the ten-eleven translocase (TET) family of DNA demethylases. Certain methylated genes, like phenyl-ethanolamine N-methyltransferase (PNMT), linked to neuroendocrine differentiation, may play a role in *SDHx*-mutated PGL development [20]. Moreover, hypermethylated genomic regions often lead to the silencing of tumor suppressor genes promoting cancer development.

Succinate dehydrogenase (SDH) converts succinate to fumarate as part of the TCA cycle and catalyzes the reduction of ubiquinone to ubiquinol as part of the electron transport chain. The biochemical phenotype of *SDHx* tumors is characterized by the hypersecretion of dopamine (and its metabolite methoxytyramine) alone or dopamine and norepinephrine. *SDHx* mutations are accompanied by intra-adrenal tumors less commonly. *SDHC* and *SDHD* mutation-associated PGLs are predominantly located in the head and neck region compared to *SDHB* PGLs that are commonly found in the abdomen [21,22]. The prevalence of malignancy is higher in *SDHB*-related PPGLs (~30%) compared to *SDHA*, *SDHC* and *SDHD* (incidence of metastasis ~0–4%). The high metastatic risk of *SHDB*-related PPGLs still remains largely unclear despite extensive research [23].

SUCLG2, a GTP-specific succinyl-CoA synthetase, is a subunit of SUCL succinyl-CoA ligase (SUCL), an enzyme that provides substrate for succinate dehydrogenase (SDH; mitochondrial complex II [CII]). Recently, *SUCLG2* has been identified as a novel candidate gene in hereditary PPGL. In the work of Vanova et al. 2021, *SUCLG2*-mutated PPGL tumors and *SUCLG2*-deficient chromaffin cells revealed the decrease in the level of the SDHB subunit of SDH, and faulty assembly of the complex II, resulting in aberrant respiration and elevated succinate accumulation [24].

Fumarate hydratase (or fumarase) catalyzes the hydration of fumarate to L-malate in the TCA cycle. Heterozygous germline mutations in *FH* gene are associated with hereditary leiomyomatosis and renal cell cancer (HLRCC) syndrome and less commonly hereditary PPGL [25].

Germline mutations in malate dehydrogenase 2 (*MDH2*) have been linked to PPGL tumorigenesis and malignant PPGL development. *MDH2* encodes the mitochondrial malate dehydrogenase, which converts malate to oxaloacetate. Accumulation of malate inhibits the α-ketoglutarate dependent enzymes, including HIF-prolyl hydroxylases, and DNA and histone demethylases, leading to a pseudohypoxic and hypermethylator phenotype, oncogenic pathway activation and PPGL formation [26].

Recently, a truncating germline isocitrate dehydrogenase 3B (*IDH3B*) mutation was found in a patient with PGL. Isocitrate dehydrogenases IDH1 and IDH2 are NADPH-dependent enzymes catalyzing the reversible oxidative decarboxylation of isocitrate to α-ketoglutarate. Unlike IDH1 and IDH2, IDH3 is NAD-dependent and catalyzes the irreversible conversion of isocitrate to α-ketoglutarate. IDH3B mutated tumor samples showed an elevated α-ketoglutarate/isocitrate ratio, which resulted in a hypermethylator phenotype [27].

In another recent work it was shown that *SLC25A11* encoding the mitochondrial alpha-ketoglutarate/malate carrier is a novel paraganglioma susceptibility gene. The loss-of-function germline mutations in the *SLC25A11* gene decrease alpha-ketoglutarate levels and alter the alpha-ketoglutarate/succinate ratio, which is associated with hypermethylator phenotype and metabolic reprogramming. Moreover, *SLC25A11* mutations are strongly associated with the development of metastatic PPGL [28].

Glutamic-oxaloacetic transaminase 2 (GOT2) is a mitochondrial enzyme, which catalyzes irreversible transamination of aspartate and alpha-ketoglutarate to form oxaloacetate and glutamate and is also involved in stimulating the malate/aspartate shuttle. Gain-of-function mutation in *GOT2* gene was recently reported in a patient with metastatic PGL. This mutation showed an elevated succinate/fumarate ratio similar to that observed for *SDHx* PPGLs [27]. This mutation increases intracellular aspartate level as well, which promotes cancer cell proliferation [29].

Mutations in the dihydrolipoamide S-Succinyltransferase encoding *DLST* gene lead to the accumulation of 2-hydroxyglutarate which similarly to succinate and malate is considered to be an oncometabolite by inhibiting the α-ketoglutarate dependent enzymes (the HIF-prolyl hydroxylases, DNA and histone demethylases). PPGL associated with *DLST* mutations expressed similar expression and methylation profile to *EPAS-1* related PPGL [30].

Under normoxic conditions, the oxygen-dependent EGLN1 (also known as PHD2) enzyme containing the prolyl hydroxylase domain (PHD) binds a hydroxyl group to the HIF-1α and HIF-2α (also known as EPAS1) subunit. This signal is recognized by VHL (Von Hippel–Lindau) protein, which targets the HIF-1α and HIF-2α subunit for proteasomal degradation. Under hypoxic conditions, HIF-1α and HIF-2α accumulate and form a heterodimer with HIFβ, which translocates to the nucleus and functions as a transcription factor [17]. HIF activates the transcription of genes that affect multiple cellular processes and play a role in the process of tumor formation, invasion and survival [31,32]. A common feature of gene mutations associated with the pseudohypoxic pathway is that they induce a hypoxic response under normoxic conditions during constitutive activation of the HIF pathway.

Mutations in the *VHL*/*EGLN1*/*EPAS1* pathway are 25% hereditary with moderate metastatic risk [4,17]. Germline mutations in the *VHL* gene cause Von Hippel–Lindau disease, which associates with hemangioblastomas, clear cell renal cell carcinomas, and pheochromocytomas [33]. Of all apparently sporadic PPGL patients, 1% to 13% have germline *VHL* mutations, [4]. The pheochromocytomas in VHL patients are most commonly intra-adrenal [22,34]. Germline or somatic loss-of-function mutation of *EGLN1* or *VHL* leads to stabilization of HIF-2α, which causes increased transcription of HIF target genes affecting angiogenesis, proliferation and migration.

### 2.2. Kinase Signaling Cluster

PPGL belonging to this cluster exhibit mostly benign, well differentiated, mostly epinephrine secreting tumors that predominantly occur in the adrenal gland. Germline mutations are present in 20% of cases. Often multiple tumors are present especially in association with the germline mutations of *RET* gene, but *MAX* and *TMEM127* mutations are associated with multiplex neoplasms as well. Approximately 50–60% of PPGL belong to this cluster. [4]. Unlike cluster 1, these tumors have an intact phenyl-ethanolamine N-methyltransferase (PMNT) function, therefore the epinephrine (Epi)/norepinephrine (NE) levels are elevated. In this cluster syndromic PPGLS are developed due to the activating germline mutations of proto-oncogene *RET*, *MERTK*, *MET* or the deactivating mutations of tumor suppressor *NF1*, *MAX, MYCN* and *TMEM127*.

Mutations of a tyrosine kinase receptor encoding *RET* gene lead to the continuous activation of the ERK/MAPK pathway. *RET* mutations associate with the multiplex endocrine neoplasia type 2 (MEN2) syndrome, which is characterized by the development of medullary thyroid cancer, parathyroid tumors and PCC [35,36]. Three different clinical presentations can be developed based on the mutated codons of the *RET* gene. MEN2A syndrome associated *RET* mutations results in the ligand-free homodimerization of the RET receptor which activates the PI3K-AKT-mTOR, RAS-RAF-MAPK and JUN kinase pathways. MEN2B-associated mutations affecting the tyrosine kinase domain cause ligand-independent activation of the RET receptor even in monomeric form [36,37,38,39]. The third manifestation is characterized by the sole presence of familial medullary thyroid carcinoma and in terms of pathogenesis it is a variant of MEN2A syndrome. However, the classical categorization of a kindred as FMTC is difficult. In order to not miss PPGL in small kindreds, the diagnosis of FMTC requires more than 10 carriers in the family, multiple carriers or affected members over the age of 50 years, and an adequate medical history, particularly in older members [40]. Others proposed that FMTC can be established in families with four or more cases of MTC in the absence of PCC or parathyroid adenoma/hyperplasia [41,42,43].

Germline activating mutation of *MERTK* (also known as c-Mer) has been described in a single patient with MEN2-like phenotype (medullary thyroid carcinoma and PCC) and recurrent and metastatic PGL without the presence of germline mutation in the coding and splicing regions of *RET* or any other PPGL driver gene [44]. *MERTK* encodes a tyrosine kinase receptor, which similarly to RET, activates the Raf-MEK-Erk and PI3K-Akt-mTOR pathways among others, and has been associated with various cancers [45,46].

The third tyrosine kinase receptor which is associated with inherited PPGL is encoded by the *MET* gene. Mutations of *MET* also lead to the continuous activation of the encoded tyrosine kinase receptor and therefore the activation of RAS/MAPK pathway. Germline *MET* mutations in association with PPGL are rare, but somatic mutations of *MET* commonly contribute to disease progression [47].

The *NF1* gene encodes the neurofibromin tumorsuppressor which inactivates the GAP and RAS proteins. The inactivating mutations of *NF1* lead to the well-characterized neurofibromatosis type 1 syndrome (café-au-lait spots, axillary/inguinal freckling, neurofibromas, Lisch nodules, optical gliomas, skeletal abnormalities and increased risk for certain tumors) where the penetrance of PPGL is relatively low [48,49,50].

The *MAX* gene encodes a basic helix-loop-helix (bHLH) zipper protein which forms a complex with the MYC transcription factor. Most mutations affect the bHLH region, inhibiting the binding of the MAX to other proteins-including the MYC. The inactivating mutations result in the overexpression of MYC associated genes. The MYC–MAX complex regulates more than a thousand genes that regulate the growth, life span and morphology of the cells and the cell cycle [51]. Familial PCCs in association with germline *MAX* mutations are often bilateral (in approx. 50%) but somatic mutations are documented, as well [52,53,54]. Interestingly, *MAX* mutation-related PPGL exhibit low PNMT expression which results in a noradrenergic phenotype with normal or slightly elevated metanephrine levels [55].

Germline mutations of *TMEM127* are a rare cause of familial PPGL syndrome. The endomembrane protein *TMEM127* acts as a negative regulator of mTOR pathway. Inactivating mutations of *TMEM127* lead to the decreased phosphorylation and the consecutive activation of mTOR signaling [56,57,58]. PCC is present in one-third of the germline *TMEM127* mutation carriers who develop bilateral tumors in 33% of the cases [54,58,59].

*KIF1B* mutations in PPGL have been described both somatic and germline [60]. However, as these variants are rare, further studies are required to clarify their role in PPGL development, as recent reports have questioned its involvement in PPGL tumorigenesis [61].

## 3. Somatic Alterations

### 3.1. Pseudohypoxia Cluster

The solely somatic gain-of-function mutations of *HIF-2α/EPAS1* stabilize the HIF-2α protein under normoxic conditions, which similarly to *VHL* and *EPAS1* mutations leads to neovascularization, proliferation and cell migration. Somatic mutations of *EPAS1* have been linked to PPGL formation [62,63]. Germline *EPAS1* mutations were reported in a family associated with recurrent PPGL but loss of heterozygosity or additional somatic mutations were not confirmed at the somatic level. Therefore, it is suspected that germline *EPAS1* mutations are more predisposing than causative factors in PPGL tumorigenesis [64].

Isocitrate dehydrogenases IDH1 (cytoplasmatic enzyme) and IDH2 (mitochondrial, TCA cycle-related enzyme) are reported to be exclusively somatically mutated in PPGL. *IDH* mutations lead to a neomorphic function, catalyzing the reduction of α-ketoglutarate to 2-hydroxyglutarate, leading to the pseudohypoxic and hypermethylator phenotype described in the previous section [65].

Mosaicism in cluster 1-related PPGL was reported in *EPAS1*, *VHL* and *SDHB* genes [66].

### 3.2. Kinase Signaling Cluster

In addition to the previously listed both germline and somatic mutated PPGL genes, there are some exclusively somatically mutated ones.

Continuous activating mutations of tyrosine kinase receptor *FGFR1* and mutations of the tyrosine kinase *HRAS* and *BRAF* activate the RAS/Raf/MAPK/ERK pathway. Pathogenic variants of these genes in association with PPGL were documented exclusively as somatic mutations [44,47,67,68,69,70,71]. Mutations of *HRAS* in sporadic PPGL are common, whereas *FGFR1* mutations are uncommon and *BRAF* mutations are rarely present [72].

### 3.3. Wnt Signaling Cluster

To date, only two genes, the *CSDE1* (cold shock domain E1) and *MAML3* (mastermind like transcriptional coactivator 3), have been identified and included in this purely sporadic group. Somatic mutations of *CSDE1* and somatic gene fusions of *UBTF* (Upstream Binding Transcription Factor) and *MAML3* have been reported in a few cases and might be responsible for approx. 5–10% of sporadic PPGL. The PNMT function is intact, therefore elevated Epi/NE ratios can be observed [7].

Somatic gene fusions of *UBTF-MAML3* lead to protein gain of function, which leads to DNA hypomethylation and the activation of Wnt and Hedgehog signaling pathways. The fusion genes were reported in a subset of patients with aggressive phenotype and without germline mutation in PPGL driver genes [7,73].

*CSDE1* encodes a pluripotent tumor suppressor which participates in cell-type specific apoptosis and differentiation and plays an important role in mRNA stability [74]. As *CSDE1* is located at 1p13.2 chromosomic region it could serve as a potential target for 1p deletion observed in PPGL in addition to *SDHB* loss [7].

## 4. Potential Role of Novel Genes and Clusters

With the emergence and fine-tuning of next-generation sequencing techniques, novel genes and clusters are regularly proposed and described in association with PPGL tumorigenesis.

The Cancer Genome Atlas (TCGA) describes a small fraction of PPGL presenting a fourth cluster that bears a cortical admixture signature [7]. This proposition is currently debated as it is questioned whether tumor samples could have been contaminated with non-tumoral tissue. As *MAX*-mutated PPGL were also assigned to this cluster, it raises further questions to be clarified in the future [4]. Additional studies are necessary in order to validate whether it is a unique cluster or just an artifact related to tissue processing.

Recently, the RNA exonuclease 2 protein encoding *REXO2* has been suggested as a potential PPGL susceptibility gene [75]. However, as the study described a single family and tumor tissue was not available for assessing allelic loss or loss of protein expression, careful consideration is recommended.

## 5. Disease-Modifying Factors and Somatic Mosaicism

As the current classification for malignant PPGL is based only on the presentation of distant metastases, eager attempts are made to identify potential disease modifying genetic alterations that could predict disease progression and may serve as cornerstones for the currently limited therapy [13].

Actin-dependent motor Myosin Vb gene B (*MYO5B*) encodes a motor protein which interacts with Rab-GTPases. Somatic mutations of *MYO5B* were identified in SDH-deficient tumors and are reported to accelerate tumor progression [76,77]. Similarly to *MYO5B*, the somatic mutations of *MYCN* gene have been reported in malignant PPGL harboring germline *SDHB* mutation [76]. *MYCN* is a member of the MYC family of transcription factors and encodes the N-myc proto-oncogene protein which regulates various pathways affecting cell survival, tumorigenesis, tumor progression and metastasis development [78].

Genes involved in the process of chromatin remodeling are described to affect PPGL tumorigenesis: Somatic inactivating mutations of *ATRX* have been reported to drive tumor progression in clinically aggressive PPGL [47,79]. The histone 3.3 encoding *H3F3A* somatic mutations [44] and the mostly somatic, occasionally germline mutations of the histone methyltransferase encoding *KMTD2* [80], have been associated with PPGL tumorigenesis.

Genes related to cancer development have also been linked to PPGL. Both the mutations of cell cycle regulator *CDK2NA* and the *TP53* were reported in PPGL tumors on the somatic level [71].

Somatic mosaicism refers to the presence of a genetically distinct cell population within an organism, hence the potential presence of a pathogenic variant in PPGL genes occurring during the early postzygotic development. Somatic cases have been identified more often since the introduction of NGS technology into genetic diagnostics. Due to its nature, mosaicism can lead to attenuated/partial phenotype of a disease. Indeed, in PPGL, in addition to germline and somatic mutations, mosaicism has been reported in several genes including *EPAS1, H3F3A, VHL* and *SDHB* [44,64,66,81,82]. In mosaic cases the well-described genotype–phenotype correlations are not absolutely reliable, similarly to the prediction of other manifestation of the disease. Mosaicism represents a significant difficulty regarding genetic diagnostics and clinical management as the interpretation of the relevance of such variants are challenging.

## 6. Clinical Relevance of Genetic Background of PPGL

The basis of the clinical relevance of genetic background of PPGL is the well-known genotype–phenotype association described in detail elsewhere [1,18].

From a clinical point of view alterations of the PPGL genes can be divided into two groups: 10 genes (*RET*, *VHL*, *NF1*, *SDHD*, *SDHAF2*, *SDHC*, *SDHB*, *SDHA*, *TMEM127*, *MAX)* that currently have well-defined genotype–phenotype correlations that can be used in the clinical approaches and the group of other, continuously emerging genes without soundly established genotype–phenotype associations [1,18].

After the PPGL diagnosis is verified, germline genetic testing is recommended due to the high hereditary background [6]. The genetic diagnosis (identification of the alterations of the 10 clinically relevant genes) further guides the tailoring of imaging studies and subsequent medical management based on the well-known genotype–phenotype associations [1]. Thus, PPGL diagnosed as part of complex genetic tumor syndrome, gene-specific further medical investigations and surveillance protocols are recommended (see details in [1]). Examples include medical test and surveillance for additional manifestations of the syndromes in case of pathogenic variants of *RET* or *VHL* genes; MR imaging of the abdomen in case of *MAX* and *TMEM127* pathogenic variants, MR imaging of the skull base and neck in case of *SDHC* and *SDHAF2* pathogenic variants, while MRI of the skull base and neck, thorax, retroperitoneum, and pelvis are all recommended when a pathogenic alteration in *SDHA*, *SDHB* or *SDHD* genes is found.

However, when pathogenic gene variants in the other group of PPGL genes are detected, hereditary predisposition is established, but in the lack of well-described genotype-phenotype correlations, only general medical testing, surveillance and family screening can be proposed [1].

The genetic background influences the initial and follow-up biochemical testing, imaging and therapeutic options [1]. The diagnostic and therapeutic relations of PPGL genetics are continuously developing similarly to molecular pathology with several important discoveries summarized below.

## 7. Using the Genetic Background for Individualized Patient Management

As novel genes and pathomechanisms are described yearly, it becomes evident that uniform diagnostic and therapeutic approaches are not sufficient for each patient affected by PPGL. Germline mutations affect the location and number of tumors, the biochemical characteristics, the risk for malignancy and, therefore, the overall prognosis [83]. Cluster differentiation yields the possibility to personalize the diagnostic and therapeutic approaches. As this has been extensively reviewed in literature [84], this paragraph briefly summarizes the most important hallmarks of the different clusters and how they can be exploited in the clinical setting.

### 7.1. Implications of Genetic Background for Individualized Diagnostic Approach in Metastatic PPGL

The diagnosis of malignant PPGL is only established by the detection of distant metastasis [6]. Various types of predictive systems have been suggested in order to predict metastatic disease [85]. In terms of genetic background, germline *SDHB* mutations predispose to higher risk of malignancy [6]. In limited cohorts, mutations of *FH* [25], *MAX* [86], *SDHD* [87], *SLC25A11* [28], *TERT* [88,89] and gene fusions of *MAML3* [7] have been reported to be more prevalent among malignant PPGL compared to benign tumors. Somatic mutations of *ATRX* are also associated with aggressive phenotype and this represents an independent risk factor for the clinical progression of PPGL [79,90]. Regardless of genotype, for primary and metastatic tumor localization, computed tomography (CT) and magnetic resonance imaging (MRI) are used as an initial step.

The awareness of the pathological background of different PPGLs affects the diagnostic imaging [91]. For the initial screening of *SDHx* mutation carriers, for recurrent cases, tumor staging, metastatic tumors and for the initial localization of occult or ectopic tumors, radionuclide-based functional imaging studies are recommended [5,92]. These can be ^123^I-metaiodobenzylguanidine (MIBG), 6-^18^F-fluoro-L-dopa (^18^F-FDOPA), ^18^F-fluorodeoxyglucose (^18^F-FDG), and somatostatin receptor (SSTR) scintigraphy with either using PET technology [gallium-68 DOTATATE (^68^Ga-DOTATATE)] or traditional γ-camera [technetium-99m-tektrotyd (^99m^Tc-tektrotyd) and indium-111-octreoscan (^111^In-octreoscan)].

The choice of the nuclear imaging method and of the PET tracer is influenced by the genetic background, the localization and the behavior of PPGL [93,94,95]. Cluster 1A *SDHx* mutation related PPGL exhibit strong somatostatin receptor 2 (SSTR2) expression therefore functional imaging with SSTR analogue ^68^Ga-DOTATATE positron emission tomography-computed tomography (PET/CT) offers the most sensitive functional imaging method for the diagnosis and screening of these PPGLs [96,97,98,99]. ^18^F-FDG-PET imaging for *SDHx*-related PPGL offers the second most sensitive method especially for rapidly progressive or metastatic disease [13,96,100,101,102]. ^18^F-FDOPA uptake is shown uniquely in parasympathetic HNPGL, therefore, ^18^F-FDOPA-PET may be used for *SDHD*-related HNPGLs [96,103,104,105]. For *FH* mutation related PPGL ^18^F-FDOPA-PET is likely to be the most sensitive method but as germline *FH* mutations are associated with malignant phenotype, ^18^F-FDG-PET should be considered as a second option [96,106].

Cluster 1B *VHL*, *EPAS1, PHD1/2*-related PPGLs show a stronger L-type amino-acid transporter compared to SSTR2 expression, thus, ^18^F-FDOPA imaging is more sensitive in these tumors [96,107,108].

Among cluster 2 PPGL, MEN2 and NF1 syndrome-associated cases should undergo ^18^F-FDOPA-PET or ^123^I-MIBG imaging [100].

^123^I-MIBG scan is mandatory for candidate patients for ^131^I-MIBG therapy [13]. It has to be noted, however, that in contrast to ^68^Ga-DOTATATE-PET and ^18^F-FDG-PET, MIBG scintigraphy yields lower sensitivity for the diagnosis of *VHL* and *SDHx* mutation-related PPGL and metastatic lesions [13,109,110,111] and prediction of MIBG uptake based on the genetic background of PPGL alone is difficult [112].

### 7.2. Universal and Individualized Therapeutic Approach in Metastatic PPGL

The first line of therapy for PPGL remains the surgical resection with preoperative alpha blockade in order to reduce the catecholamine-related symptoms [6,113]. Alpha blockade is recommended before systematic therapies for disseminated disease as well [13] and should be continued for at least 3 days after both ablative or systemic therapies [13]. Cortical sparing resection is recommended in all hereditary cases due to the high risk or bilateral tumor development and low risk for malignancy which is typically descriptive for cluster 2 tumors [6].

The indication for non-surgical therapies includes irresectable tumors, high tumor burden or metastatic disease. The options consist of radiotherapy, radiofrequency or cryo ablation, tumor embolization or systematic radionuclide therapy and cyclophosphamide–vincristine–dacarbazine (CVD) or temozolomide chemotherapy [13]. In the largest retrospective study, CVD treatment for metastatic PPGL yielded 37% radiographic partial response and 40% biochemical response [114]. In retrospective studies, temozolomide treatment also showed radiographic and biochemical response to therapy [115,116].

MIBG exploits the expression of the norepinephrine transporter for recycling and uptake of amines via a sodium-dependent cell membrane noradrenaline transporter (NET) into the cell. MIBG serves as a substrate for this enzyme and the accumulation of this radiopharmaceutical is taken advantage of both in imaging (^123^I-MIBG) and therapy (^131^I-MIBG), as well. In a prospective registrational trial (NCT00874614) for hypertensive metastatic MIBG-avid PPGL treated with High-Specific-Activity ^131^I-MIBG, 25% of the patients showed at least 50% reduction in all antihypertensive medication for at least 6 months and tumor size reduction was achieved in 23%, whereas stable disease was the case in 69% of the patients [117]. Additionally, despite the expected worse prognosis for patients with lung or liver metastasis, similar survival was observed when compared to those patients who did not have such metastasis [117].

**Figure 1:**
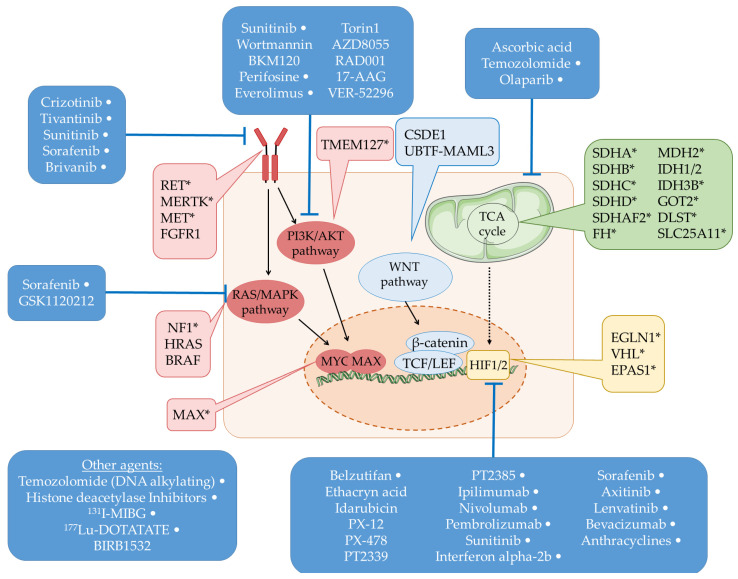
Schematic representation of the main pathways associated with PPGL tumorigenesis. The genes marked with ‘*’ are germline susceptible PPGL genes as well. The therapeutic agents targeting the associated genes or pathways are also displayed. The therapeutic agents marked with ‘White dot’ represent the specific agents which are involved in clinical trials or approved in the clinical setting, whereas the unmarked agents are being studied in in vitro or in vivo models currently.

Recently, novel, often cluster and molecular signature-specific therapeutic agents have emerged as promising options for the treatment of metastatic PPGL (Figure 1).

### 7.3. Targeted Therapy for Patients with Cluster 1 Genetic Alterations

As the hallmark of Cluster 1 PPGL is the stabilization of HIF under normoxic conditions, an evident treatment option would be HIF inhibition for these PPGLs. A HIF-2 antagonist, belzutifan, was recently approved as a treatment option as it showed promising results in case of *VHL* mutated and clear cell renal carcinoma, and is currently being evaluated regarding PPGL treatment [118].

It has been shown that *SDHB*-mutated PPGLs exhibit dysregulation in oxygen metabolic pathways (pseudohypoxia and increased reactive oxygen species); it has been suggested that targeting the redox balance pathway could be a potential therapeutic approach [119]. Indeed, a recent study reported that targeting SDHB was a promising therapeutic strategy for *SDHB*-mutated PPGL [119]. Namely, the use of pharmacologic ascorbic acid successfully suppressed SDHB-low metastatic lesions and prolonged overall survival preclinical animal model with PPGL allografts [119].

PPGLs of different clusters exhibit different metabolite profiles. In contrast to sporadic and *RET* mutation associated PPGL, Cluster 1A related PPGL with impaired TCA cycle exhibit lower levels of lactic acid [120], and increased glutamine/glutamate metabolism to support cellular anabolism [121]. A mitochondrial enzyme, glutaminase-1 (GLS-1), was a target of SDH-related studies: *SDHB* knockout cells are more sensitive to GLS-1 inhibitors [122,123]. Increased GLS-1 expression in PPGL was associated with more aggressive phenotype and shorter survival [122,124]. The glutaminase inhibitor telaglenastat (CB-839) is currently being studied within the confines of SDH-associated gastrointestinal stromal tumors and non-gastrointestinal stromal tumors as well (NCT02071862). In vitro studies of telaglenastat have demonstrated that the loss of both the respiratory complex I and II is required for effective suppression of proliferation and cell survival [125]. Complex I and II are parts of the mitochondrial electron transport chain which participates in the oxidative phosphorylation. Deciphering the molecular aspects of these tumors will yield novel targets for therapy in the future.

Pseudohypoxia deregulates the cellular energetics which prevent immune system recognition. Namely pseudohypoxia leads to T cell dysfunction, impairs tumor infiltration by T cells and induces resistance to cytotoxic T cells. Ipilimumab, nivolumab and pembrolizumab and currently evaluated as potential therapeutic options to address this phenomenon [126].

As previously mentioned, alongside HNPGLs irrespective of genetic background, *SHDx* mutation related PGLs show increased SSTR-2 and -3 expression supporting the role of SSTR-targeted therapies for these tumors [127,128]. ^177^Lu-DOTATATE treatment was recently approved by the US Government Food and Drug Administration following a phase 2 trial with promising results [117]. ^177^Lu-DOTATATE therapy should be considered for MIBG negative but SSTR-PET-positive patients with malignant PPGL. Compared to MIBG therapy, ^177^Lu-DOTATATE offers milder side effects and the reports on biochemical and radiographic response are encouraging [129,130,131,132].

### 7.4. Targeted Therapy for Patients with Alterations of Tyrosine Kinase Signaling Cluster

Tyrosine kinase inhibitors with antiangiogenic potential (axitinib, cabozantinib, dovitinib, lenvantinib, pazopanib and sunitinib) are reported with limited but promising preliminary data for treatment of disseminated PPGL [133]. The potential roles of *MET, FGFR1* and *TERT* inhibitors in PPGL have also been raised and have been tested in clinical trials [84,134].

Pathogenic variants of *TMEM127* result in an increase in the mTOR signal that may promote the occurrence of PCC, and the degree of its activation may be related to tumor invasion and metastasis. This gives the rationale of the application of another, commonly used targeted therapeutic approach, mTOR inhibition (everolimus) in PPGL [84,134].

Targeted therapy has many advantages (e.g., fewer side effects, longer survival time, and, consequently, better quality of life) in many tumor types. As neuroendocrine tumors, including PPGL, are generally considered chemo- and radioresistant tumors, targeted therapies in this field hold great promise. It is important to highlight that despite many new discoveries related to PPGL-targeted treatments, the resulting data are still limited and, in many cases, experimental. However, in the near future, they will provide a reference for clinical targeted therapy for PPGL.

## 8. Conclusions

In the last two decades, as the extraordinary diverse genetic background of PPGL has unfolded, these tumors have become the center of attention for studies in genetics, oncology and biochemistry. These studies are the flagships of translational medicine: the emergence of the high-throughput next-generation sequencing methods has led to the identification of numerous PPGL genes and redefined the clinical genetic screening of these patients and their families; these were closely followed by the studies deciphering the tumorigenesis which yielded novel potential prognostic factors, diagnostic and therapeutic options. Although these tumors are rare, their diversity highlights that even an infrequent tumor should not be treated uniformly. As our knowledge increases, the next decades certainly will be spent under the aegis of personalized and precision healthcare for the patients affected by these tumors.

## Figures and Tables

**Table 1:** Cluster differentiation of the currently known PPGL susceptibility genes.

		Germline and Somatic	Exclusively Somatic
Cluster 1: Pseudohypoxic cluster	Cluster 1A	*SDHA/B/C/D/AF2*, *FH*, *MDH2, IDH3B*, *GOT2*, *DLST*, *SLC25A11*	*IDH1/2*
	Cluster 1B	*EGLN1*, *VHL*	*EPAS1*
Cluster 2: Kinase signalling cluster		*RET, MERTK*, *MET*, *NF1*, *MAX*, *TMEM127*	*FGFR1*, *HRAS*, *BRAF*
Cluster 3: Wnt signaling cluster		-	*CSDE1*, *UBTF-MAML3*
